# Learning to diagnose using patient video case in paediatrics: perceptive and cognitive processes

**DOI:** 10.1007/s40037-012-0026-z

**Published:** 2012-09-27

**Authors:** Thomas Balslev

**Affiliations:** Centre of Medical Education, Brendstrupgaardsvej 102, 8200 Aarhus N, Denmark

**Keywords:** Patient video cases, Collaborative learning, Contextual learning, Authentic learning

## Abstract

Thomas Balslev, a paediatric neurologist and educational researcher, defended his thesis on 24 November 2011. The thesis included five published papers, and investigated learning with authentic, brief patient video cases. With analysis of a video case in a small group, learning processes and sharing of knowledge was intensely stimulated. Small group discussion and subsequent listening to an expert’s think-aloud were particularly effective approaches to enhance diagnostic accuracy among non-experts. In a descriptive study, expertise-related differences during analysis of patient video cases were characterized, and in a controlled study, different types of visual modelling were tested.

## Introduction

In paediatrics, authentic patient video recordings (PVCs) are frequently used for diagnostic purposes as well as for in-context learning. Examples are patients with epileptic seizures or abnormal movements. Such symptoms may be periodic or episodic, and may be too infrequently seen by the learner in real life. This thesis aimed to study how PVCs might improve learning, and also aimed to show what clinicians attend to in PVCs and what they think while they analyze them. A final study studied how visual guidance in PVCs might benefit learning.

## How resident’s learning processes may be improved

We used a randomized, controlled before-and-after design with two groups of residents. After initial analysis of an identical text vignette, one group analyzed a video case, the other analyzed a text case. Learning processes were audio recorded and analyzed. A stimulated recall procedure was applied to get an in-depth analysis. Learning processes and also sharing of knowledge among participants was intensely stimulated by the video case.

## How interactive approaches may improve diagnostic accuracy among non-experts

A stepwise, interactive teaching approach with patient video cases was designed to identify approaches particularly useful to help non-experts learn from experts. This study showed that participation in small groups and subsequent listening to an expert’s think-aloud were particularly effective approaches. Learning to diagnose by video cases can be improved by the interactive participation of junior as well as senior clinicians.

## How visual expertise differs with duration of experience

In this study we wished to identify expertise-related differences during analysis of PVCs. We used eye tracking to examine the perceptive processes and a concomitant think-aloud procedure to examine the cognitive processes. Not surprisingly, more experienced clinicians had a high diagnostic accuracy. In addition, more experienced clinicians spent more time looking at relevant areas, and less time searching other areas. At the same time, experienced clinicians were able to invest more of their cognitive capacity in active building and evaluation of diagnostic hypotheses.

## How visual expert modelling in PVCs may benefit learning

This randomized controlled study was done to examine the effect of visual modelling by superimposing an expert model’s eye movements on the PVCs during teaching. Three different teaching videos were produced: control, circle display and spotlight display. Medical students taught by spotlight teaching videos were faster and more focused in their visual search when subsequently analyzing test videos. It therefore appears that visual modelling by teachers can benefit learning.

